# Effects of encapsulated rosemary extract on oxidative and microbiological stability of beef meat during refrigerated storage

**DOI:** 10.1002/fsn3.1258

**Published:** 2019-11-11

**Authors:** Seyede Salimeh Rashidaie Abandansarie, Peiman Ariaii, Mehdi Charmchian Langerodi

**Affiliations:** ^1^ Department of Food Science & Technology Sari Branch Islamic Azad University Sari Iran; ^2^ Department of Food Science & Technology Ayatolla Amoli Branch Islamic Azad University Amol Iran; ^3^ Department of Agricultural Extension and Education Sari Branch Islamic Azad University Sari Iran

**Keywords:** beef meat, lipid oxidation, microbial spoilage, nanoencapsulation, rosemary

## Abstract

In this study, the effect of rosemary extract in two free and encapsulated forms to increase the shelf life of beef meat during a 28‐day refrigerated storage period was investigated. For this purpose, rosemary was extracted using different extraction methods including ultrasound, solvent, and supercritical fluid extraction. The amount of phenolic compounds, antioxidant properties (free radical scavenging capacity of DPPH radical, ferric reducing antioxidant power), and antimicrobial activity of rosemary extract against pathogenic bacteria were evaluated. According to the results, the highest amount of phenolic compounds, antioxidant, and antimicrobial activity was observed in rosemary extracted by ultrasound method that used for next study (*p* < .05). In order to encapsulation of the rosemary extract, basil seed gum and soybean protein isolate separately and in combination form (1:1 w:w ratio) were used as carriers. Based on the particle size, zeta potential, and encapsulation efficiency tests, the best carriers were soybean protein isolate that used as a carrier for encapsulation. Then, to investigate the effect of rosemary extract to increase the shelf life of beef meat, 5 treatments including control, rosemary extract with concentrations of 800 ppm and 1,600 ppm, and nano‐capsulation form of it with 800 ppm and 1,600 ppm concentrations were selected and they were periodically evaluated for chemical and microbial analysis (peroxide value, Thiobarbituric acid, color index, pH, and total viable count). The results showed that rosemary extract has an antimicrobial and antioxidant properties which could increasingly delay microbial spoilage and lipid oxidation of beef meat fillets, nano‐capsulation form of rosemary could increase these qualities. The best results were observed in nano‐capsulation of rosemary extract with 1,600 ppm (*p* < .05) as well as increased the shelf life of fillets till 21st day. Therefore, it seems that encapsulated rosemary extract could be used as a natural preservative in beef meat and meat products.

## Graphical abstract

Meat and meat products have a high corruption capability due to high moisture and lipid content and rich in protein and minerals. Using of antioxidant and antimicrobial preservatives in meat and meat products is very common to prevent the lipid oxidation and spoilage and increased the shelf life and quality of products. The results of this study showed that rosemary extract with a concentration of 1,600 ppm significantly delayed the microbial and lipid oxidative during storage in beef meat fillets and increased the shelf life of fillets to 21st day. Therefore, it seems that encapsulated rosemary extract could be used as a natural preservative in beef meat and meat products.

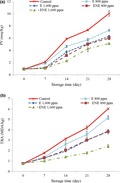

## 1 INTRODUCTION

Meat and meat products have a high corruption capability due to high moisture and lipid content and rich in protein and minerals. The most common chemical changes in meat products are lipid oxidation. Lipid oxidation is a complex process and depends on the chemical composition of meat, access to light, oxygen, and storage temperature, and finally, it causes undesirable changes in the sensory properties (color, texture, and flavor) and the nutritional quality of meat (Devatkal, Thorat, & Manjunatha, 2014; Shah, Bosco, & Mir, 2014). Using antioxidant and antimicrobial preservatives in meat and meat products is very common to prevent the lipid oxidation and spoilage and increased the shelf life and quality of products. Currently, consumers considering the harmful effects of chemical and synthetic food preserves were eager to use the natural preservatives derived from plant, animal, and microbial resources that in addition to increase the shelf life of food products and also protect from the harmful effects of chemical preservatives (Burt, 2004). Plants have been considered as the source of natural antioxidants due to their effective compounds such as polyphenolic compounds, flavonoids, tannins, and phenolic acids. In addition to the antioxidant properties, these compounds also have antimicrobial, anticancer, and antimutagenic properties (Dawidowiez, Wianowska, & Baraniak, 2006).

One of the herbal extracts that have an antimicrobial and antioxidant properties is rosemary extract. The Rosemary (*Rosmarinus officinalis* L.) is a plant belonging to the Lamiaceae family. This herb is always green and very fragrant. The origin of this plant is Mediterranean region and also growth along the Mediterranean coast to Asia. This plant has valuable and fragrant materials. The most important of these compounds include tannin, bitter materials, phenolic compounds, and flavonoids (El‐Rajoob, Massadeh, & Omari, 2008; Jiang et al., 2011).

There are several methods for extraction from plant, such as mass transfer, supercritical fluid extraction, ultrasound, subcritical water extraction, and microwave. Modern methods extracted the effective compounds in shorter time with less solvent content. In ultrasound method, penetration of solvent into plant tissue is well done and this method compared to other methods has a higher efficiency and speed. Therefore, effective cellular degradation and mass transfer are main factors that increased the efficiency by ultrasound method (Chen, Liu, Chiu, & Hsu, 2016).

Since plant extracts have highly active compounds, they may lose beneficial effects through exposure to oxygen or during processing. Therefore, it is necessary to use specific methods to protect them to achieve the highest antioxidant activity.

Microcapsulation or encapsulation is a technology for putting different materials, liquids, solids, and gases into a homogeneous or heterogeneous coating (Nedovic, Manojlovic, Levic, & Bugarskib, 2011). Usually carbohydrates, lipids, and proteins were used as walls or capsules with a micro and nano particle size (dimensions ranging from 1 to 100 nm). Soybean protein isolate is one of the most popular vegetable protein sources that have a wide application in the formulation of food products and it can also be used directly as a food supplement. Globulins glycinin and b‐conglycinin are the main components of soybean protein isolate. These globes have different structure and functional characteristics (Robert et al., 2010). Therefore, the purpose of this study was to investigate the antioxidant effects of free and encapsulation forms of rosemary extracts to increase the shelf life of beef meat during refrigerated storage.

## 2 MATERIALS AND METHODS

### 2.1 Preparation of rosemary plant

Aerial parts of rosemary plant were collected from the Sari city located in Mazandaran province, in spring season, dried in shadow, and powdered plants.

### 2.2 Extraction of rosemary by ultrasound

About 10 g of rosemary powder samples was put in 100 ml of ethanol:water (50%:50%) at 45°C for 20 min in an ultrasound bath at 20 KHz. Then, the solutions were filtered through Whatman filter paper No. 1 and solvents evaporated by vacuum evaporation. The extract was stored at −18°C until next study (Maleki, Ariaii, & Fallah, 2016).

### 2.3 Extraction by solvent

Rosemary powder with ethanol–water solvent (50%:50%) was placed at a room temperature (1:10 w/v), in dark room for 48 hr. After smoothing and evaporation of solvent by rotary evaporator, the extract was stored in a dark glass container until use at −18°C (Maleki et al., 2016).

### 2.4 Supercritical fluid extraction

Supercritical fluid extraction (SFE) was done according to method described by Delfanian, Esmaeilzadeh Kenari, and Sahari (2015). For this purpose, 10 g of rosemary powder was mixed with ethanol solvent (1:10) as a modifier. Extraction was done by supercritical carbon dioxide equipment (Suprex MPS/ 225) at 35°C, 100‐bar pressure for 30 min.

### 2.5 Determination of phenolic compounds

#### 2.5.1 Total phenolic compounds

The total phenolic compounds of rosemary extract were measured according to the Folin–Ciocalteu procedure described by Donald, Prenzler, Autolovich, and Robards (2001) and expressed as gallic acid equivalents (GAE) in milliequivalents per gram dry material. The basis of this work is to restore the Folin representation by phenolic compounds in an alkaline environment to create a blue complex that shows the maximum absorption at 760 nm.

### 2.6 Evaluation of antioxidant properties

#### 2.6.1 Free radical scavenging capacity of DPPH radical

The antioxidative activity rosemary extract was elucidated by 2, 2‐diphenyl‐1‐ picryhydrazyl (DPPH) free radical scavenging capacity of the extracts (Maleki et al., 2016). DPPH solution (0.004% w/v) was prepared in methanol. About 1 ml of rosemary extract was added to a sample solution (0.1 ml, 1 mg/ml in methanol). After 30 min, absorbance at 517 nm was measured and the percentage of radical scavenging activity was calculated from the following equation:

% Radical scavenging = (1 − Abs. sample/Abs. control) × 100 Abs. control is the absorbance of the DPPH solution without sample and Abs. sample is the absorbance of the control samples.

### 2.7 Determination of ferric reducing antioxidant power

The ferric reducing antioxidant power (FRAP) was measured according to method described by Benzie and Strain (1999). In this method, antioxidants play the regenerative activity role, leads to the recovery of iron III into iron II. Depending on the revitalization power of the extract, the solution color changes to green or blue.

### 2.8 Bactericidal assay

In vitro bactericidal activity of rosemary extract was examined versus pathogens including *Staphylococcus aureus* (PTCC1431), *Pseudomonas aeruginosa* (PTCC 1074), and *Escherichia coli* (PTCC 1399). These bacterial strains were obtained the from Faculty of Veterinary Medicine, Tehran University, Tehran, Iran, which were prepared from lyophilized stocks.

### 2.9 Determination of minimum inhibitory concentration and minimum bactericidal concentration values

Minimum inhibitory concentration (MIC) test for selected bacteria was carried out by the method recommended by NCCLS. At first, 0.1 ml of rosemary extract was placed into tubes containing 1 × 10^8^ CFU/ml of the above‐mentioned bacteria strains. Then, all the samples were incubated at 37°C for 24 hr. The MIC value is defined as the lowest concentration of the rosemary extract at which the bacteria do not demonstrate any visible growth. Also, the lowest concentration in which there were no bacteria defined as the minimum bactericidal concentration (MBC) value.

### 2.10 Encapsulation of rosemary extract

Encapsulation of rosemary extract was done using the method that recommended by Chranioti, Nikoloudaki, and Tzia (2015). In order to encapsulate the rosemary extract, basil seed gum and soybean protein isolate separately and in combination form were used as carriers. Solution was kept in refrigerator for 24 hr. Rosemary extract was dissolved in dichloromethane/methanol solution (1:2 w/w) and mixed with Basil seed gum and soybean protein isolate (as wall materials) with 1:1 ratio. The pH of mixtures adjusted on 7.4 with phosphate buffer and then mixed with a magnetic stirrer (12.000 rpm, 5 min, and 10°C). Finally, the encapsulation form of rosemary extract was dried by a freeze dryer at a pressure of 0.017 millipascal at −57.7°C for 48 hr.

### 2.11 Approval of encapsulation form of rosemary extract

#### 2.11.1 The encapsulation efficiency

The encapsulation efficiency (%) of polyphenols was determined according to the method described by Jivan, Yarmand, and Madadlou (2014) using the following formula (Jivan et al., 2014):

EE% = (Ce/Ct) × 100where Ce is the content of polyphenols released from capsules (ppm), and Ct is the polyphenols content added into the particle formation solution (ppm). In this way, 0.6 g of powder with 20 mL of alkaline water (10.5 ppm) was mixed with a magnetic mixer and then stirred for 30 min. Then, it was centrifuged at 4,000 ×*g* for 10 min. The supernatant phase was reached to pH: 7.

The average diameter, dispersion index and particle size distribution, and particle‐specific surface were measured by Zetasizer (Nano zs). The samples were diluted with deionised water with 5:1 ratio. The zeta potential was measured by particle electrophoresis using the same instrument. Samples were equilibrated at 25°C prior to analysis (Joye, Davidov‐Pardo, & McClements, 2015).

### 2.12 Sample preparation

Approximately 30 kg femur of beef meat with average weight of 900 ± 50 g was prepared and transported to laboratory in ice boxes. Transmission and filleting (cut into pieces of 6 × 6 × 4 cm) were immediately done in 1 hr. Then, the meats were washed by potable water in a laboratory.

### 2.13 Preparation of beef meat samples and chemical analyses

In this assay, 5 treatments including control, rosemary extract with concentrations of 800 ppm and 1,600 ppm, and nano‐capsulation form of rosemary extract with 800 ppm and 1,600 ppm concentrations were selected. Samples immersion in mention solutions containing free extract and NC solution for 60 min, and then, packed in polyethylene bags, labeled, and stored at 4°C for 28 days. Different chemical and microbiological analysis was carried out at intervals of 7 days. All experiments were carried out in triplicate.

### 2.14 Measurement of peroxide value

Peroxide value (PV) was measured according to the AOCS method (AOCS, 1980). PV was calculated and expressed as milliequivalent peroxide per kg of sample:

$$\text{PV}\;\left(\text{meq/kg}\right)=\left(S\;\times N\right)/W\times 1,000$$

where, S is the volume of titration (ml), N the normality of sodium thiosulfate solution (*N* = 0.01), and W the sample weight (kg) (AOCS, 1980).

### 2.15 Determination of thiobarbituric acid reactive substances

The thiobarbituric acid (TBA) assay was determined according to the method described by AOCS, (1980). TBA value was expressed as milligram malonaldehyde (MDA) equivalents per kilogram of fish muscle.

### 2.16 pH measurement

About 5 g of each sample was added to 45 ml of distilled water and placed in a mixer for 30 s. Then, the pH of the samples was measured using a digital pH meter (Sallam, 2007).

### 2.17 Determination of fillet color

The fillets samples were selected to evaluate the color parameters (L, a*, and b*). Visible color parameters were measured by a Colorflex Hunter Lab colorimeter (Hunter Lab Inc) (Stadnik & Dolatowski, 2011). The color parameters were L* (luminosity) for lightness, ranging from 0 for black to 100 for white; a* (redness) for red/green and b* (yellowness) values for yellow/blue.

### 2.18 Microbiological analyses

Total viable count (TVC) was determined by inoculating 0.1 ml of the sample homogenate onto triplicate sterile plates of dried Tryptic Soy Agar (Merck) using the surface spread technique, then the plates were incubated for 48 hr at 37°C (Sallam, 2007).

### 2.19 Statistical analysis

The obtained data were subjected to one‐way analysis of variance using SPSS statistical software, release 20.0. Duncan's new multiple range test (at the 95% level) was performed to determine the significant differences of the means at the 5% probability level (*p* < .05). All experiments were carried out in triplicate.

## 3 RESULTS AND DISCUSSION

### 3.1 Phenolic compounds

Phenolic compounds in fruits and vegetables, because of high potential antioxidant activity and prevent the releasing of free radical, give a lot of attention and approval by many researchers, (Hajimahmmodi et al., 2012). In the present study, the amount of total phenolic compounds of rosemary extract was between 434.1 and 699.13 μg/g dry weight. In the Afonso et al. (2013) study, the amount of phenolic compounds of rosemary aqueous extract was 166.7 μg/g and in Perira, Pinheiro, Heldet, and Moura (2017) study was 409.1 μg/g. The amounts of phenolic compounds in the present study were higher than those reported that may be related to the environmental and genetic parameters, and conditions of harvesting rosemary, as well as differences in extracting method and kind of solvents (Delfanian et al., 2015).

According to the results, the phenolic compounds of rosemary extract obtained by ultrasound method were significantly higher than the other extraction methods (699.13 μg/g dry weight) (*p* < .05), the phenolic compounds in the supercritical fluid extraction treatment were 5,665.37 μg/g dry weight and the lowest amount of it was observed in water–ethanol treatment (410.4 μg/g dry weight) (*p* < .05). The reason for higher phenolic compounds in ultrasound method may be related to the cavitation phenomenon during ultrasonic process. In fact, ultrasound waves facilitate both the extraction process including swelling of the tissue, as well as the removal of the compounds from it by creating porosities and pores in the cell wall, so, living cells that affected by these waves were destroyed and releasing their substances be better and easier (Kadam, Tiwari, Smyth, & Donnell, 2015). The supercritical fluid extraction after the ultrasound method was able to extract more phenolic compounds than the solvent method. Extraction of high‐polarity material such as phenolic compounds is one of the disadvantages and limitation of the supercritical fluid extraction method (Delfanian et al., 2015).

### 3.2 The antioxidant properties of rosemary extract

**Figure 1:**
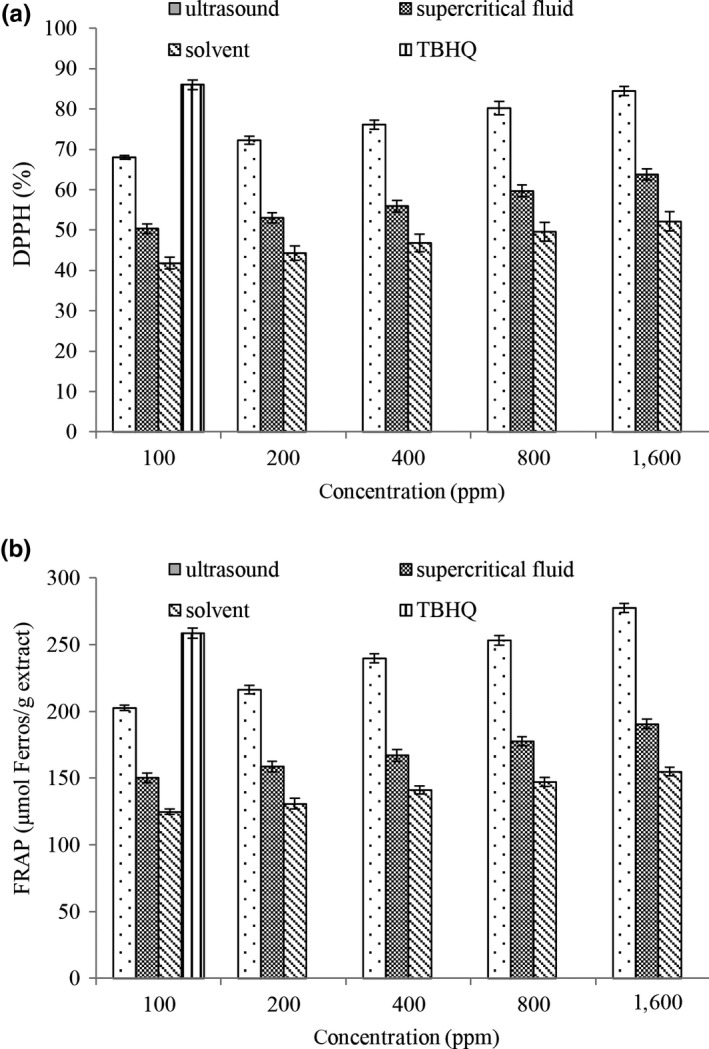
Effects of extraction methods and extract concentration on the antioxidant activity (DPPH [a] and ferric reducing antioxidant power [b])

The results of this study showed that the free radical scavenging capacity (DPPH) was increased by increasing the concentration of rosemary extract (Figure 1a) (*p* < .05). Herbal extracts have antioxidant activity due to their phenolic compounds and have high capacity for donating hydrogen, electron, and free electron (Delfanian et al., 2015). Also, rosemary extract obtained by ultrasound method showed a more effective efficiency in free radical scavenging capacity (DPPH) compared with other extraction methods (*p* < .05). This result can be related to the higher ability of the ultrasound method to extract effective compounds including phenolic compounds with high antioxidant properties that increased the antiradical activity. The results of this study were consistent with the Maleki et al. (2016) finding in relation to free radical scavenging capacity (DPPH) by *Cichorium intybus *L extract.

The results of the current study showed that in all concentrations, the highest and the lowest in the ferric reducing antioxidant power (FRAP) were in ultrasound extraction and solvent methods, respectively (Figure 1b) and rosemary extracted by ultrasound method had a higher antioxidant activity than TBHQ at 1,600 ppm (*p* < .05). This reduction agent can be considered as polyphenols in the rosemary extract, especially phenolic compounds which are the dominant composition with antioxidant activity (Yousfbeyk et al., 2014). In this study, rosemary extract was a potent antioxidant in both antioxidant tests. The best results were observed in rosemary treatment extracted by ultrasound method. Also, antioxidant properties of rosemary extract have been reported by other researchers (Afonso et al., 2013; Perira et al., 2017).

### 3.3 Minimum inhibitory concentration and minimum bactericidal concentration values

**Figure 2:**
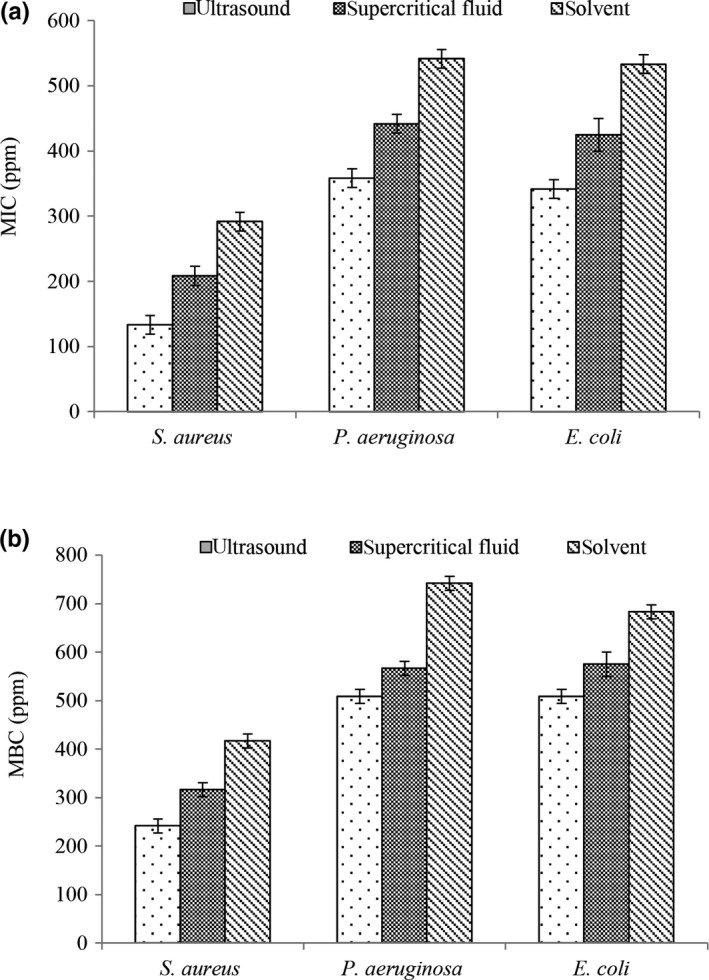
Effects of extraction methods and extract concentration on the antibacterial activity (MIC [a] and MBC [b])

The results of this study showed that rosemary extracted by ultrasound has higher antimicrobial activity on selected bacteria (Figure 2). According to the results of MIC (Figure 2a) and MBC (Figure 2b), *S. aureus* had the lowest resistance among pathogenic bacteria and *P. aeruginosa* was the most resistant pathogen bacteria. Several reports have suggested that gram‐positive bacteria are more sensitive to antibacterial compounds compared with gram‐negative bacteria. This reason may be due to absence of lipopolysaccharide in cell wall of gram‐positive bacteria which prevented the entry of active compounds into the cytoplasmic membrane (Bozin, Mimica‐Dukic, Samojlik, & Jovin, 2007). After antioxidant and antimicrobial tests, rosemary that extracted by ultrasound method was selected for further testing.

### 3.4 Encapsulation analysis

Particle size and particle size distribution are of the importance factors to determine the properties of colloidal systems. The values and stability of these parameters play a significant role in determining the stability of the colloid carrier system and encapsulation efficiency. According to the results (Table 1), particle size of rosemary extract coated by the basil seed gum significantly was larger than other treatments (154.9 nm), and also, the particle size in encapsulated form coated by soybean protein isolate was smaller than other treatments (12.48 nm) (*p* < .05). The smaller particle sizes have more stability, which due to higher resistance to gravity in smaller sizes (Fathi, Mozafari, & Mohebbi, 2012). Razavizadeh, Kadkhodaee, and Zaferani (2015) investigated the effects of different compounds on the particle sizes of nanocapsules, they stated that the type of ingredients had an effect parameter on particle size. The dispersion index of nanoparticles theoretically limited to zero to one, and values greater than 0.5 represent a large dispersion of particle size (Tamjidi, Shahedi, Varshosaz, & Nasirpour, 2013). In this study, the dispersion index of nanoparticles was <0.15 (Table 1), which shows a monotone distribution and confirmed the efficiency of encapsulation by different carriers.

**Table 1:** Results of encapsulation extracts analysis

Treatment	Particle size (nm)	Dispersion index	Zeta potential (mv)	Efficiency (%)
Basil seed gum	154.69 ± 4.63^a^	0.14 ± 0.01^a^	18.40 ± 0.72^c^	58.71 ± 3.20^c^
Basil seed gum–soybean protein	139.52 ± 4.19^b^	0.12 ± 0.009^ab^	20.33 ± 0.89^b^	61.35 ± 0.84^b^
Soybean protein isolate	126.48 ± 3.69^c^	0.11 ± 0.009^b^	22.49 ± 1.05^a^	66.39 ± 1.87^a^

^a,b,c^ Different small letters in the same column represents significant difference (*p* < .05).

According to the results, the dispersion index in rosemary extracts coated by basil seed gum was higher than other treatments (0.14). Also, the dispersion index in rosemary extracts coated by soybean protein was lower than other treatments (0.11) (*p* < .05) which indicated that the soybean protein isolate was more suitable. Measuring the zeta potential is a useful assay to control the massification and sedimentation of nanoparticles as important factors for sustainability. In general, in colloid depression, zeta potential systems with a 30+ to 30 mv are sustainable (Mozafari, Johnson, Hatziantoniou, & Demetzos, 2008). According to the results of zeta potential, rosemary extracts coated by the Basil seed gum were less than other treatments (18.48 mV) and zeta potential in rosemary extracts coated by soybean protein was more than other treatments (22.49 mV) (Table 1) that shows the higher stability of the nanoparticles produced by this carrier. According to previous studies (Rasti, Jinap, Mozafari, & Yazid, 2012; Sebaaly, Greige‐Gerges, Agusti, Fessi, & Charcosset, 2016) similar to our study, by decreasing the particle size, zeta potential was increased.

Regarding the results, the encapsulation efficiency of rosemary extracts coated by the Basil seed gum was less than other treatments (58.71%) and encapsulation efficiency in extracts that coated by soybean protein isolate was higher than other treatments (66.39%; *p* < .05). According to the results, soybean protein isolate used as carrier and rosemary extract in free form and nanoencapsulation forms with 800 and 1,600 ppm were used in beef meat.

### 3.5 Chemical and microbial changes of beef meat during refrigerated storage

#### 3.5.1 Peroxide value (PV) changing

**Figure 3:**
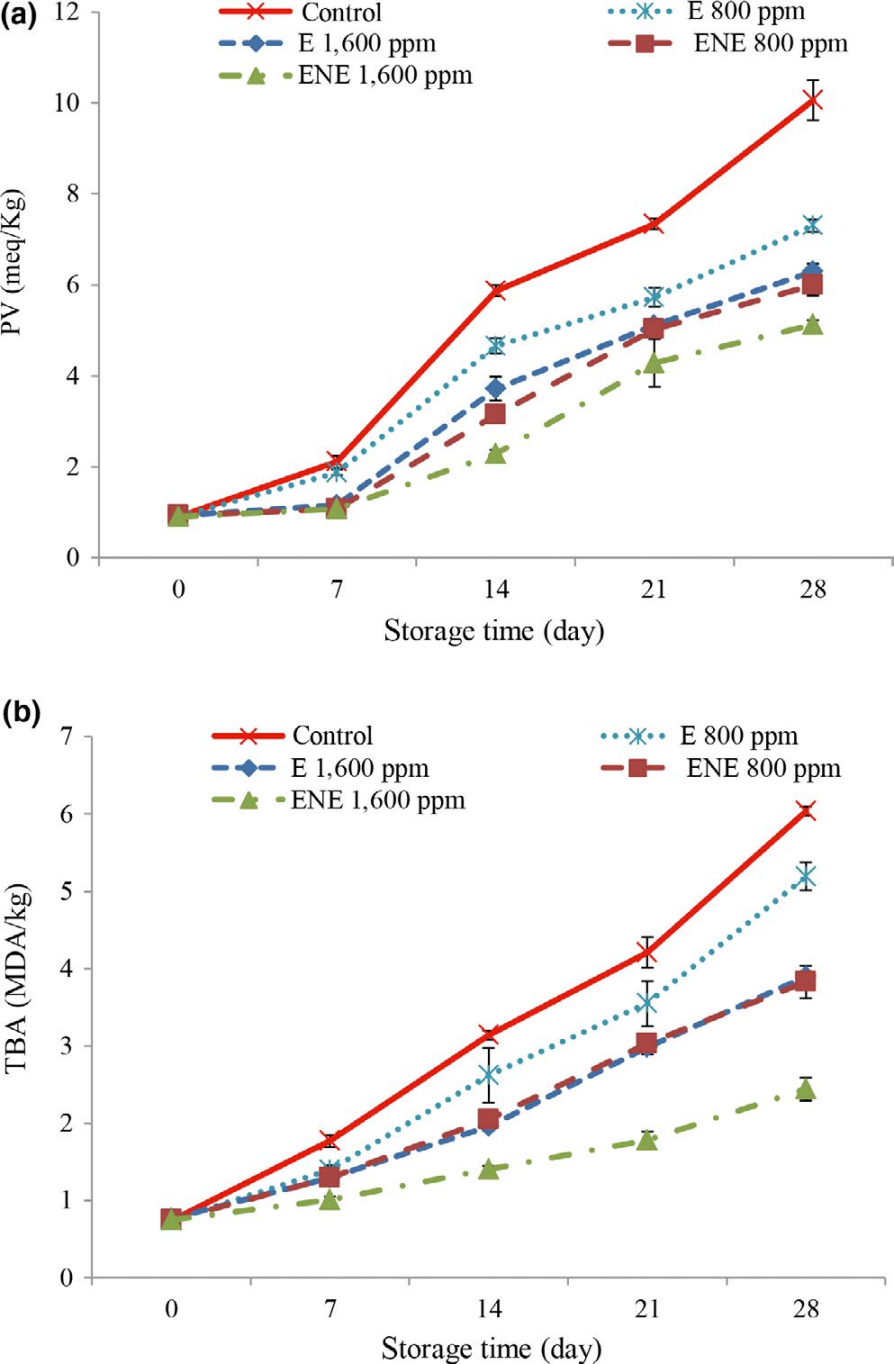
Changes in peroxide value (a) and thiobarbituric acid (b) of different treatment during storage

The results of PV (Figure 3a) showed that by increasing time, the PV increased in all treatments. According to the results of statistical analysis, the highest peroxide value was observed in control treatment. Lower values of PV in treatments containing rosemary extract are due to the antioxidant properties of rosemary extract. The antioxidant activity of rosemary extract is related to phenolic compounds including rosmarinic acid*,* followed by carnosic acid and carnosol (Tavassoli & Emamjomeh, 2012) which has the ability for chelating metal ions and neutralize reactive oxygen species (ROS). Several studies have reported that the antioxidant effect of natural extracts is dependent on the amount of antioxidant compounds (Bagheri, Izadi Amoli, Tabari Shahndash, & Shahosseini, 2016; Javadian, Shahoseini, & Ariaii, 2017). In the 28th day, the lowest of PV was observed in the nano‐capsulation form of rosemary extract with a concentration of 1,600 ppm (12.5 milli mEq/kg) and the highest values were observed in control treatment (10.06 mEq/kg). So, encapsulation of rosemary extract increased the antioxidant properties and shelf life of beef meat during storage. Also, several studies have shown that encapsulation improved the bioactive compounds such as polyphenols (Fang & Bhandari, 2010). The acceptable level of PV for consumer is 5 mEq/kg of meat (Yanar, 2007). At the end of the storage, the peroxide value in all samples was higher than limited level, but PV only in the 1,600 ppm nano‐encapsulated treatment until the 21st day was in limited level.

### 3.6 Changes in thiobarbituric acid

The results of the changes in the TBA are shown in Figure 3b. According to the results, TBA was increased in all studied treatments during the storage. Increasing the TBA during storage can be attributed to the oxidation of lipid and production of volatile compounds in the presence of oxygen. According to the results of statistical analysis, the TBA in the control treatment most of the days was observed in maximum level. Better results were observed in higher concentrations. The compounds in the extracts are an appropriate electron and proton donator and their intermediate radicals are very stable due to the phenomenon of electrons moving in the benzene ring and the lack of a site susceptible to oxygen attack. The compounds in the rosemary extract including carnosic acid, carnosol, ressmanon, monin rosemary, and rosemary null have the potential effects to neutralize the free radicals and they also able to inhibit ions metal such as Fe^2+^, thus reducing the rate of formation of activated oxygen molecules (Mohamed & Mansour, 2012).

Also, the better results were observed in the nano‐capsulation forms of rosemary extract and in the 28th day, the lowest values of TBA were observed in the nano‐capsulation form of rosemary extract with a concentration of 1,600 ppm and the highest values were observed in control treatment. In fact, it can be expressed that the encapsulation of rosemary extract increases the antioxidant properties and shelf life of beef meat. The acceptable level of TBA for consumer is 2 MDA/kg meat (Campo et al., 2006). At the end of the storage, the TBA in all samples was higher than limited level, and the range of TBA only in the 1,600 ppm nano‐encapsulated treatment until the 21st day was in acceptable level.

### 3.7 Changes in pH values

**Figure 4:**
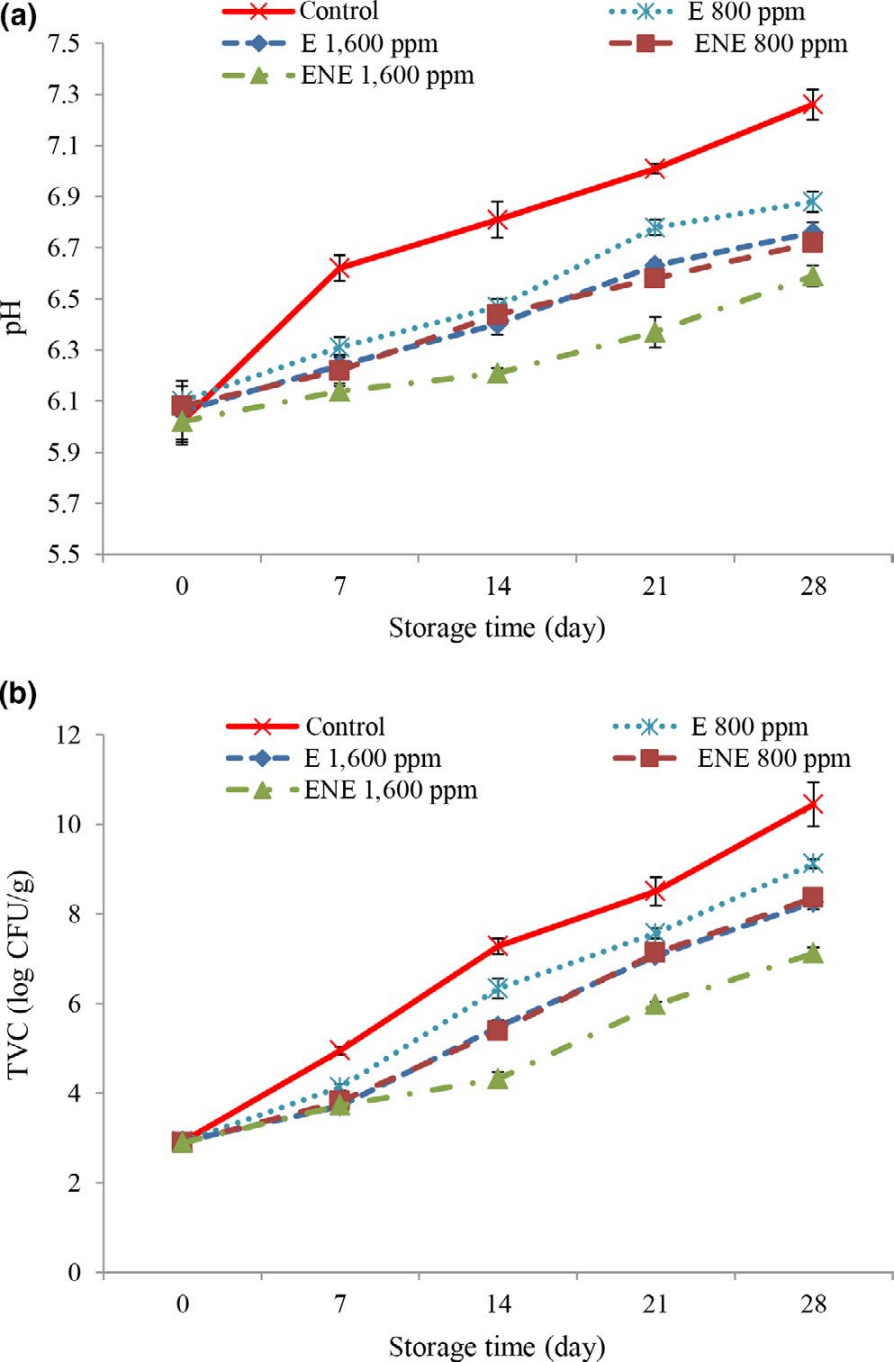
Changes in pH (a) and total viable count (b) of different treatment during storage

The results for pH changing are shown in Figure 4a. According to the results, by increasing the storage time, secondary metabolites produced by microorganisms and protein deamination were increased (Gill, 1983) and by breaking down of amino acids, ammonia was produced and accumulated in meat and finally increased the pH value. The pH value in the control treatment in most days was observed in maximum level. Better results were observed in higher concentrations. The lower pH value in the sample containing the rosemary extract can be attributed to the antibacterial effect of it. Similar results were obtained by Vilela et al. (2016) when they add rosemary and lean extract to the beef meat. The main reason for the increase of pH was the production of volatile amino acids by some spoilage microorganisms such as pseudomonads. Also, the better results were observed in the nano‐capsulation forms of rosemary extract and in the 28th day, the lowest pH value was observed in the nano‐capsulation form of rosemary extract with a concentration of 1,600 ppm, and the highest values were observed in control treatment. This changing may be related to an increased the antibacterial properties of rosemary extract after encapsulation or to maintain the antibacterial properties of rosemary extract for a longer period after encapsulation.

### 3.8 Total viable count

The results TVC changing are shown in Figure 4b. TVC in the control treatment in most days was observed in maximum level. Better results were observed in higher concentrations. The lower TVC that observed in the sample containing the rosemary extract can be attributed to the antibacterial effect of the rosemary. The lower TVC in encapsulated treatments can be due to phenolic compounds. The phenol compounds in the plant extracts destroyed the outer membrane of microorganisms, releasing the liposaccharides and increased the permeability of cytoplasmic membrane to ATP and finally, it leads to the end of cellular energy storage and death (Burt, 2004). The better results were observed in the nano‐capsulation forms of rosemary extract, and in the 28th day, the lowest TVC count was observed in the nano‐capsulation form of rosemary extract with a concentration of 1,600 ppm and the highest TVC was observed in control treatment. That may be related to increase the antibacterial properties of rosemary extract after encapsulation. Using the soybean protein isolate helps to protect bioactive compounds and food product against lipid oxidation and prevent the growth of pathogenic microorganisms (Gortzi, Lalas, Tsaknis, & Chinou, 2006). Also, increasing the antimicrobial activity of herbal extracts after nanoencapsulation has been reported by other researchers (Bagheri et al., 2016; Gortzi et al., 2006; Javadian et al., 2017). 7 log CFU/g have been suggested as an acceptable level for TVC (Hayes, Stepanyan, Allen, O’Grady, & Kerry, 2010). At the end of the storage, the TVC count in all samples was higher than standard level, and TVC range only was in limited level in the 1,600 ppm nano‐encapsulated treatment until the 21st day.

### 3.9 Changes in fillet color

**Figure 5:**
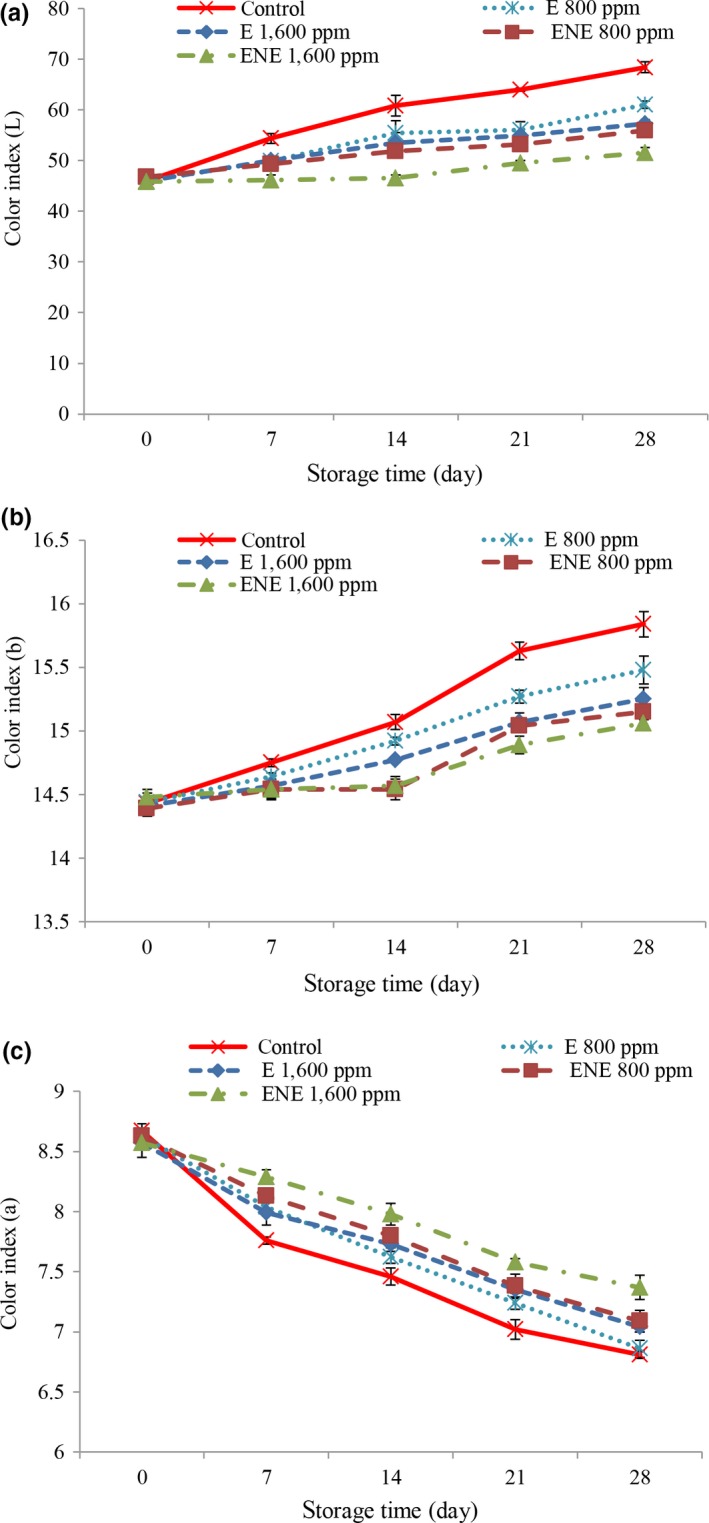
Changes in color index L, b, and a (a, b, and c respectively) of different treatment during storage

According to the results, by increasing the storage time, the color index L (Figure 5a) and b (Figure 5b) increased and a (Figure 5c) decreased. According to the statistical analysis, most of the changing was observed in control treatment in most days. Better results were observed in higher concentrations and in nanoencapsulation treatments. So, on the 28th day, the minimum changes in color index were observed in the nano‐capsulation form of rosemary extract with a concentration of 1,600 ppm and the highest changes were observed in control treatment. Nair, Kiess, Nannapaneni, Schilling, and Sharma (2015) considers that changing in meat color influencing by lipid oxidation and believes that the accumulation of products due to meat oxidation increased the oxidation of oxymyoglobin and production of methemoglobin. By reducing the speed of oxidation, we could reduce the degradation rate of color. One of the ways to reduce oxidation was to use antioxidant compounds. The extract of rosemary that used in this research is full of phenolic compounds. The phenolic acids oxidize quinines that are circular and react with lysine, cysteine, methionine, and tryptophan in the myoglobin molecule, frequently, the myoglobin polymerization was increased and the amount of heme also increased, by increasing the amount of iron in the tissue, the tissue becomes redder. Most reactive phenolic compounds are rosmarinic acid which has four phenolic groups, followed by carnosic acid and carnosol having two phenolic groups. Therefore, it was expected that by increasing the concentration of rosemary extract, the redness of the meat be increased.

## 4 CONCLUSION

In conclusion, the results of this study showed that rosemary extracted by ultrasound method has a high phenolic compounds and strong antimicrobial and antioxidant activities. Encapsulation of rosemary extract coated by soybean protein isolate increased the antimicrobial and antioxidant properties and nano‐capsulation form of rosemary extract with a concentration of 1,600 ppm significantly delayed the microbial and lipid oxidative during storage in beef meat fillets and increased the shelf life of fillets to 21st day. Using the similar encapsulated plant extracts in meat products could reduced the lipid oxidation and could be an effective way to improve the microbial status, increased the organoleptic quality of meat products and finally increased the shelf life of these products.

## CONFLICT OF INTEREST

The authors declare that they do not have any conflict of interest.

## INFORMED CONSENT

Written informed consent was obtained from all participants.
